# Still at sea: a patient’s ongoing journey with non-obstructive hypertrophic cardiomyopathy

**DOI:** 10.1093/ehjcr/ytag136

**Published:** 2026-03-02

**Authors:** Fraser C Goldie, Rachel C Myles, Caroline J Coats

**Affiliations:** School of Cardiovascular and Metabolic Health, University of Glasgow, 126 University Place, Glasgow G12 8TA, UK; West of Scotland Inherited Cardiac Conditions Service, Queen Elizabeth University Hospital, 1345 Govan Rd, Glasgow G51 4TF, UK; School of Cardiovascular and Metabolic Health, University of Glasgow, 126 University Place, Glasgow G12 8TA, UK; West of Scotland Inherited Cardiac Conditions Service, Queen Elizabeth University Hospital, 1345 Govan Rd, Glasgow G51 4TF, UK; School of Cardiovascular and Metabolic Health, University of Glasgow, 126 University Place, Glasgow G12 8TA, UK; West of Scotland Inherited Cardiac Conditions Service, Queen Elizabeth University Hospital, 1345 Govan Rd, Glasgow G51 4TF, UK

**Keywords:** Non-obstructive hypertrophic cardiomyopathy, Mavacamten, Electrocardiogram

Cardiac myosin inhibitors target hypercontractility and are effective for symptomatic obstructive hypertrophic cardiomyopathy (oHCM).^1^ ODYSSEY-HCM (NCT05582395) was a phase 3 trial testing mavacamten in symptomatic non-obstructive HCM, which did not meet its primary endpoints for improving exercise capacity or quality of life compared with placebo.^2,3^ A 71-year-old female limited by exertional dyspnoea, despite beta-blockers, participated in the study. Cardiac magnetic resonance confirmed HCM (*Figure 1A*) (LVEF 60%; no late gadolinium enhancement), and exercise echocardiogram excluded LVOT obstruction. There was evidence of left ventricular diastolic dysfunction, with mitral valve E/A ratio of 0.8 and E/E′ average of 16.9 (*Figure 1B*). Prior to enrolment, her electrocardiogram showed voltage criteria for hypertrophy with lateral T wave inversion (*Figure 1C*). NT-proBNP was 435 ng/L and clinical genetic testing found no pathogenic variants.

**Figure 1:**
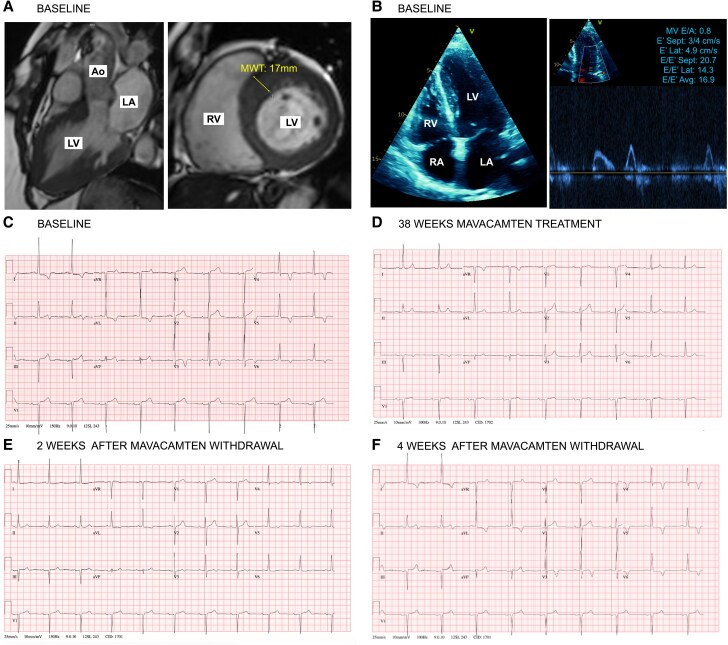
Demonstrates baseline investigations and ECG changes with mavacamten treatment and withdrawal in a patient with non-obstructive hypertrophic cardiomyopathy: cardiac MRI 3-chamber and short-axis at baseline (*A*); echocardiography 4-chamber and mitral inflow pulse wave Doppler, with annotated diastolic function parameters, at baseline (*B*); ECG at baseline (*C*); ECG after 38 weeks of mavacamten treatment (*D*); ECG 2 weeks after mavacamten withdrawal (*E*); and ECG 4 weeks after mavacamten withdrawal (*F*). Ao, ascending aorta; E/E′ Avg, Mitral valve peak early diastolic inflow velocity-to-mean early diastolic tissue velocities ratio; E/E′ Lat, Mitral valve peak early diastolic inflow velocity-to-early diastolic tissue velocities ratio at lateral mitral annulus; E/E′ Sep, Mitral valve peak early diastolic inflow velocity-to-early diastolic tissue velocities ratio at medial mitral annulus; E′ Lat, peak early diastolic tissue velocity at lateral mitral annulus; E′ Sep, peak early diastolic tissue velocity at medial mitral annulus; ECG, electrocardiogram; LA, left atrium; LV, left ventricle; MRI, magnetic resonance imaging; MV E/A, mitral inflow peak E-to-A wave velocities ratio; MWT, maximum wall thickness; RA, right atrium; RV, right ventricle.

During 36 weeks mavacamten treatment (blinded at the time), symptoms markedly improved. At a routine outpatient visit at 38 weeks, ECG normalisation was noted (*Figure 1D*) and contemporaneous echocardiogram showed reduction of E/E′ average to 9.0, with E/A ratio remaining at 0.8. Treatment continued for an additional 10 weeks until study end. After stopping mavacamten, ECG showed lateral T wave flattening at 2 weeks (*Figure 1E*) and deep T wave inversion at 4 weeks (*Figure 1F*). NT-proBNP was 196 ng/L at 2 weeks and 463 ng/L at 4 weeks. Average E/E′ rose to 19.9, with E/A ratio of 0.8. Symptoms returned with worsening dyspnoea.

Resolution of ECG changes with mavacamten has been reported in 22% real world patients with oHCM.^4^ These are attributed to reduced left ventricular wall stress, reverse remodelling, and improved myocardial perfusion as LVOT obstruction is relieved. This case provides compelling support for a similar effect of mavacamten in some patients with non-obstructive HCM. We demonstrate novel insight into ECG changes, which have never been reported in nHCM, complemented by improvements in left ventricular diastolic function as also reported in recently published literature.^3^ The evolution of ECG and biomarker changes after mavacamten withdrawal implies that T wave inversion was mediated by hypercontractility and wall stress rather than static hypertrophy. This builds upon previous literature and support the need for ongoing study of cardiac myosin inhibitors in a non-obstructive population.

## Supplementary Material

ytag136_Supplementary_Data

## Data Availability

No new data were generated or analysed in support of this research.
