# Supplementary figures and images for: Still at sea: a patient’s ongoing journey with non-obstructive hypertrophic cardiomyopathy
Source: Eur Heart J Case Rep. 2026 Mar 2;10(3):ytag136. doi: 10.1093/ehjcr/ytag136 (PMC12998440; doi:10.1093/ehjcr/ytag136)

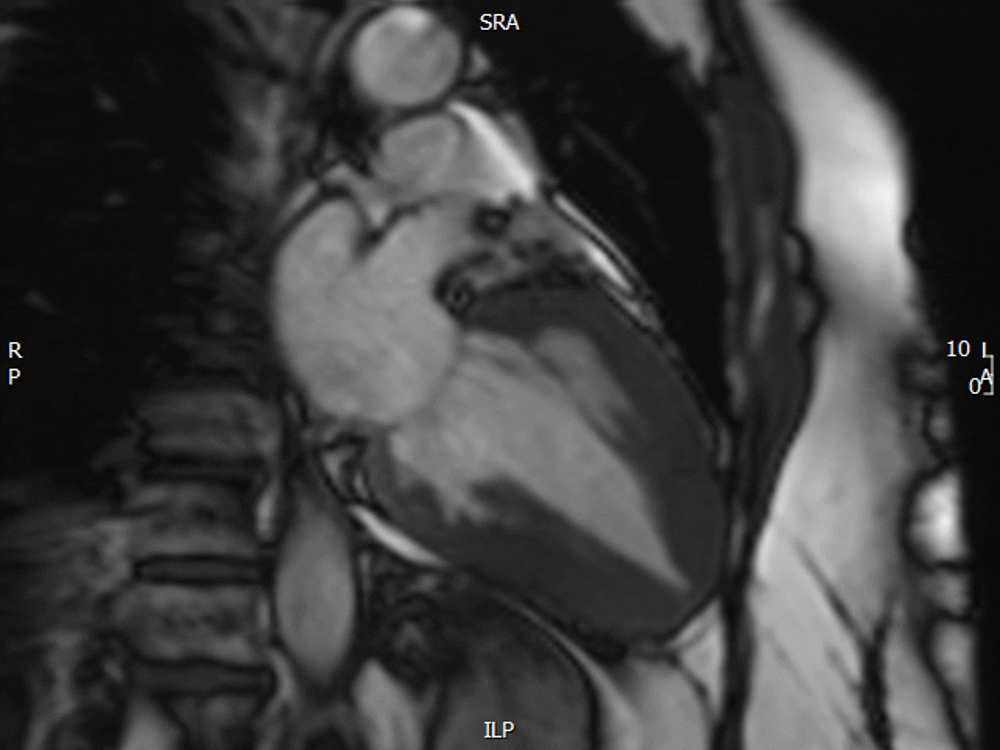

Supplement: ytag136_Supplementary_Data [file ytag136_supplementary_data.zip › Supplemental_2Ch.gif]

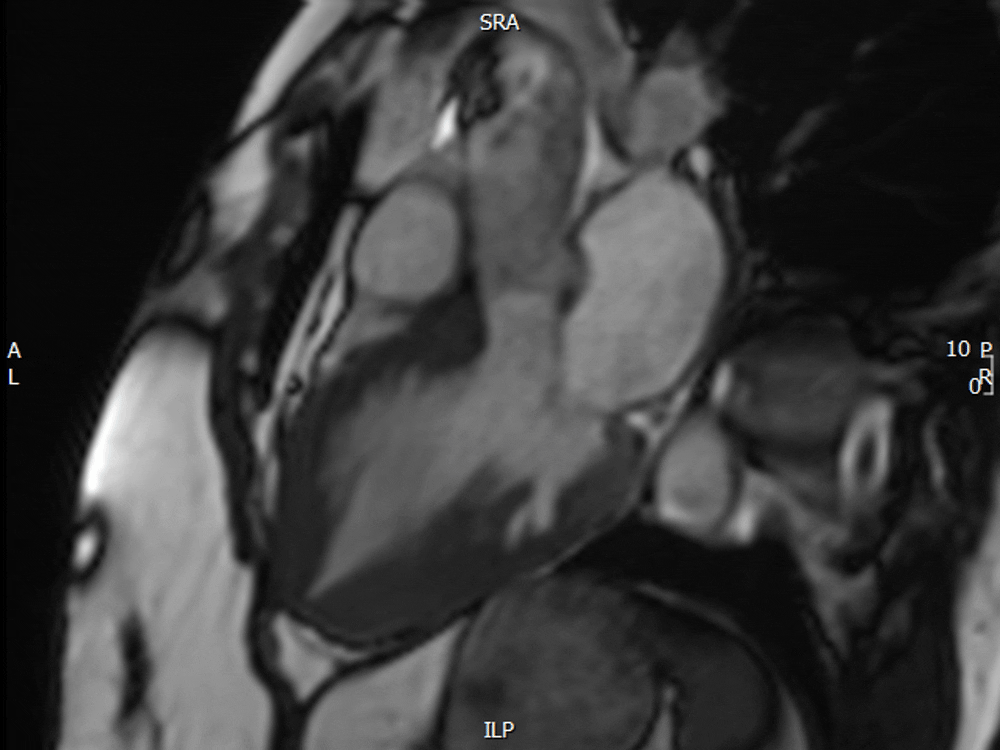

Supplement: ytag136_Supplementary_Data [file ytag136_supplementary_data.zip › Supplemental_3Ch.gif]

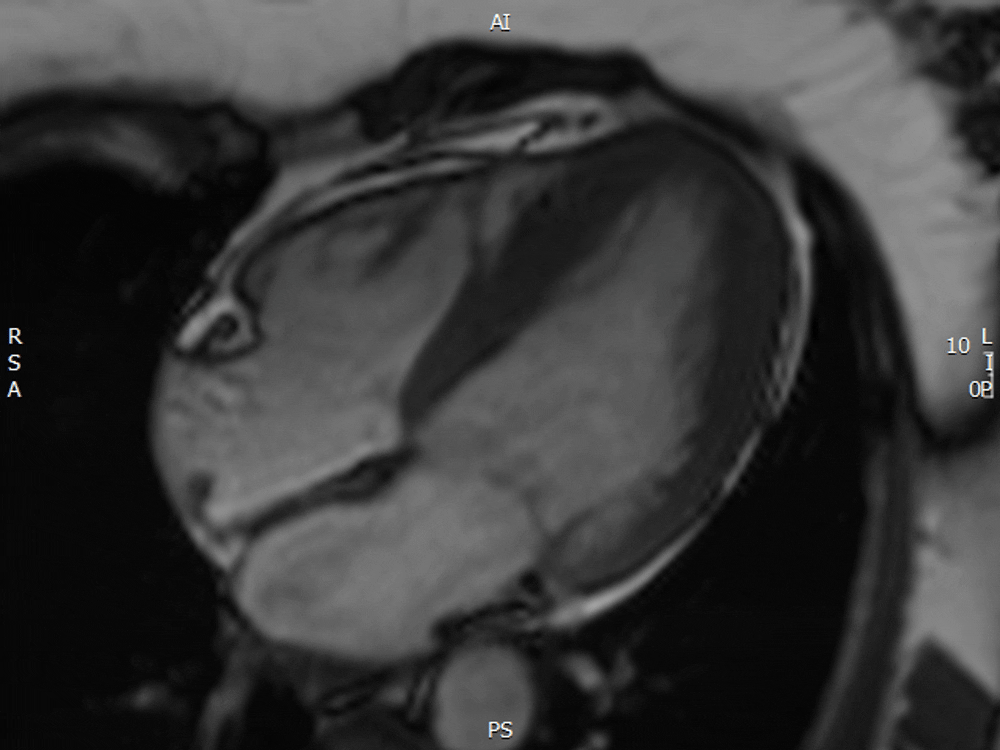

Supplement: ytag136_Supplementary_Data [file ytag136_supplementary_data.zip › Supplemental_4Ch.gif]

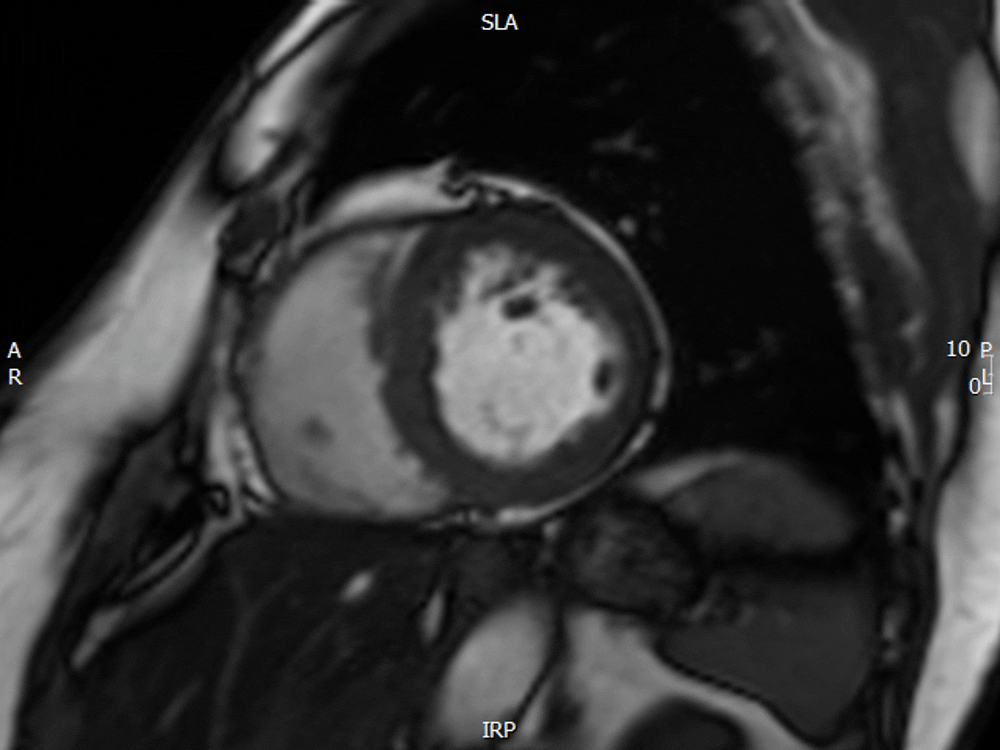

Supplement: ytag136_Supplementary_Data [file ytag136_supplementary_data.zip › Supplemental_SAX.gif]
